# Reduced CpG island methylation of the TBC1D8 gene may predict risk for osteoporosis in Chinese postmenopausal women

**DOI:** 10.18632/oncotarget.24065

**Published:** 2018-01-06

**Authors:** Qian-Qian Ma, Lv Lin, Qi Yao, Jun Yang, Yan Hu, Jing-Bo Yu

**Affiliations:** ^1^Department of Gerontology, Ningbo First Hospital, Ningbo, Zhejiang Province, 315010, People’s Republic of China; ^2^Ningbo Center for Disease Control and Prevention, Ningbo, Zhejiang Province, 315010, People’s Republic of China; ^3^Medical Examination Center, Ningbo First Hospital, Ningbo, Zhejiang Province, 315010, People’s Republic of China; ^*^These authors contributed equally to this work

**Keywords:** osteoporosis, methylation, postmenopausal women

## Abstract

Objective: In this study, we collected samples from postmenopausal women aged >60 y and evaluated bone mineral density (BMD) in addition to other biochemical variables to evaluate risk factors for osteoporosis. Furthermore, we investigated whether an association exists between the CpG island methylation levels in the promoter region of the TBC1D8 gene and osteoporosis incidence. Our goal was to identify contributing factors to the pathogenesis of osteoporosis and provide a theoretical basis for osteoporosis testing and therapy.

Materials and Methods: We used questionnaires to collect data from Chinese Han women in their communities. The following parameters were measured: uric acid, high density lipoprotein, low density lipoprotein, fasting blood glucose, serum creatinine, serum calcium, serum phosphorus, alkaline phosphatase, P1NP, β-CTX, PTH, 25(OH)D and bone mineral density from lumbar spine 1 to 4, femoral neck, and total hip. DNA was also extracted to assess the methylation level of the TBC1D8 gene.

Conclusions: Our findings suggest that a lower body mass index (BMI) infrequent exercise and certain sleep durations may be associated with osteoporosis. In addition, higher serum creatinine, β-CTX and PTH and lower 25(OH)D levels may be associated with osteoporosis. In Chinese Han postmenopausal women, decreased methylation of the TBCF1D8 gene promoter CpG islands is associated with osteoporosis. Finally, we also observed that TBC1D8 is negatively correlated with high density lipoprotein in postmenopausal women with osteoporosis.

## INTRODUCTION

Osteoporosis is a metabolic bone disease characterized by increased bone strength and increased risk for fracture. Osteoporosis is classified as either primary or secondary. Primary osteoporosis occurs in people of all ages, but most commonly in postmenopausal women and elderly men. By contrast, secondary osteoporosis occurs in response to drugs, other diseases and additional factors [1]. Osteoporosis often causes pain in the back, shortening of the body length, kyphosis, and decreased respiratory function. Patients may experience osteoporotic fractures, which not only cause physical and mental anguish but also increase economic burden. At present, osteoporosis, diabetes and cardiovascular disease are considered the three major pathologies in the elderly by the World Health Organization (WHO).

In studies of osteoporosis, increasing attention is focused on genomics. So far, more than 100 osteoporosis-related genes have been identified. Recently, some findings have demonstrated that polymorphisms in the estrogen receptor (ER) [2, 3], type I collagen (COL1) [4–6], vitamin D receptor [7, 8], and low density lipoprotein receptor-related protein 5 (LRP5) [9, 10] genes are highly associated with osteoporosis. TBC1D8 is a member of the human protein molecular activity regulation genes and is an activator of the GTP enzyme. A genome wide study combining the Framingham Osteoporosis Study that contained 7633 Caucasian women and 3657 men revealed that 2q 11.2 of TBC1D8 is a novel susceptibility loci for osteoporosis related traits [11]. Studies on single nucleotide polymorphisms in osteoporosis are relatively abundant; however, epigenetic studies examining osteoporosis are few in number. Gene methylation is a reversible chemical modification of DNA that occurs in binuclear sites of CpG islands. Methylation of CpG islands inhibits RNA polymerase or binding of interfering transcription factors to the gene gap, resulting in suppression of gene expression [12]. One study found that there were significant differences in genome wide methylation in bone cells from the femoral head region of patients with osteoporotic hip fracture and hip osteoarthritis [13]. Another article demonstrated that the methylation levels of Alu sequences are associated with the risk of osteoporosis in postmenopausal women [14]. These findings all suggest that gene methylation may be indirectly associated with osteoporosis.

As mentioned above, the TBC1D8 gene has been recently discovered to have a correlation with osteoporosis. TBC1D8 is a GTPase activator and small GTPase. As a class of single molecule proteins belonging to the GTPase superfamily, they modulate osteoclast proliferation, differentiation, and survival downstream of M-CSF and RANKL signaling [15]. In addition, small GTPases play significant roles in the nitrogenous bisphosphonates pathway to inhibit bone resorption that occurs through impaired protein prenylation [16, 17]. To our knowledge, an association between TBC1D8 gene methylation and osteoporosis has yet to be examined. We conducted this study to further study the potential pathogenic mechanisms of osteoporosis.

## RESULTS

### Clinical characteristics

We collected data from 226 Han women aged > 60 years. Among participants, we discovered 38 patients with osteoporosis, a prevalence rate of 16.8% in the cohort. At final count, 30 osteoporosis cases were conclusively identified after excluding individuals with incomplete data. To maintain a 1:1 ratio, we chose age-matched healthy controls from the participants. The clinical characteristics of these subjects are shown in Table 1. There were no significant differences with respect to age, menopause age, history of fracture, regular intake of calcium supplements, alcohol intake, afternoon naps and insomnia. In addition, all participants in the osteoporosis and control groups had no history of smoking. However, we found significant differences in BMI, exercise time and sleep duration between osteoporosis and control cases. Individuals in the osteoporosis group had lower BMI compared with those in the control group. Individuals with osteoporosis exercised for < 30 min, yet most control cases exercised for 30-60 min. For sleep duration, osteoporosis participants were concentrated in two ranges, < 6 h or >7 h, in contrast with control cases, who were mainly in the 6-7 h range.

**Table 1:** Contrast of clinical characteristics in osteoporosis and control groups

	Osteoporosis (*n* = 30)	Control (*n* = 30)	*P* value
Age (years)	71.00 ± 6.86	70.30 ± 7.29	0.661
BMI (Kg/m^2^)	22.48 ± 3.17	35.36 ± 3.10	< 0.001^*^
Age of menopause (years)	51.37 ± 3.52	50.15 ± 4.71	0.234
History of fracture	15/30	9/30	0.114
Taking calcium tablets regularly	9/30	12/30	0.417
Smoking	0/30	0/30	
Alcohol drinking	7/30	6/30	0.754
Exercise time (min/day)			
< 30	17/30	7/30	
30–60	10/30	18/30	0.031^*^
> 60	3/30	5/30	
Sleep condition Sleep duration (h/day)			
< 6	16/30	12/30	
6–7	4/30	13/30	0.030^*^
> 7	10/30	5/30	
Afternoon nap	17/30	19/30	0.598
Insomnia	13/30	10/30	0.426

^*^P < 0.05, it is significant statistical difference.

### Biochemical variables and BMD

Sixty individuals were subjected to BMD testing, and blood samples were sent for further biochemical analysis. The contrasting biochemical variables and bone mineral density levels in the osteoporosis and control groups are shown in Table 2. The results show no significant differences in calcium, phosphorus, uric acid, ALP, glucose, HDL, LDL or P1NP between the two groups. Predictably, participants in the osteoporosis group had a lower BMD compared with those in the control group. In addition, individuals in the osteoporosis group had lower creatinine, and as a result, eGFR was higher and in umbilical cord blood, 25(OH)D was lower and β-CTX and PTH were higher compared with controls. These variables were all statistically different between the osteoporosis and control groups.

**Table 2:** Contrast of biochemical variables and bone mineral density levels in osteoporosis and control groups

	Osteoporosis	Control	P value
BMD (g/cm^2^)			
L1-L4	0.80 ± 0.06	1.08 ± 0.15	< 0.001^*^
Total hip	0.69 ± 0.07	0.92 ± 0.11	< 0.001^*^
Femur neck	0.63 ± 0.07	0.85 ± 0.13	< 0.001^*^
Biochemical varaibles			
Calcium (mmol/L)	2.32 (2.26, 2.36)	2.33 (2.27, 2.38)	0.296
Phosphorus (mmol/L)	1.12 (1.11, 1.25)	1.26 (1.11, 1.33)	0.099
Uric acid (μmol/L)	272.50 (227.25, 337.75)	342.50 (242.75, 373.75)	0.093
ALP (U/L)	69 (57.75, 86.50)	70 (56.75, 76.50)	0.487
Creatinine (μmol/L)	57.50 (52.75,66)	53 (48.75, 57.75)	0.036^*^
eGFR (mL/min·1.73m^2^)	91.99 (80.41,103.64)	102.06 (91.59, 111.69)	0.027^*^
Glucose (mmol/L)	5.57 ± 1.00	5.67 ± 0.99	0.687
HDL (mmol/L)	1.39 ± 0.30	1.47 ± 0.39	0.399
LDL (mmol/L)	3.53 ± 0.76	3.39 ± 0.82	0.490
25(OH)D (ng/mL)	16.30 (12.75, 18.38)	17.65 (15.53, 20.63)	0.037^*^
β-CTX (ng/mL)	0.38 (0.29,0.48)	0.29 (0.18, 0.45)	0.034^*^
P1NP (ng/mL)	42.79 (33.38, 48.61)	33.29 (28.15, 41.48)	0.147
PTH (pg/mL)	31.22 (25.75, 37.67)	23.12 (13.79, 29.27)	0.022^*^

^*^P < 0.05, it is significant statistical difference.

### Methylation results

**Figure 1:**
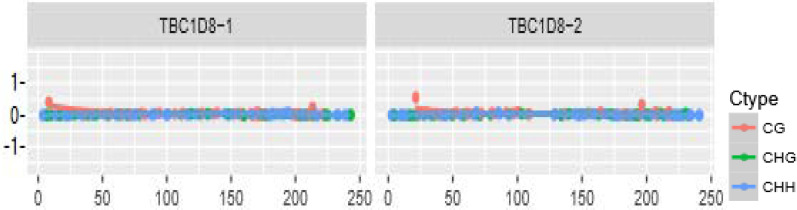
Distribution of CpG island methylation in the promoter region of TBC1D8 in the osteoporosis group.

**Figure 2:**
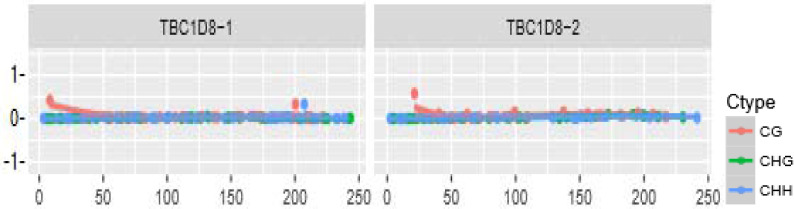
Distribution of CpG island methylation in the promoter region of TBC1D8 in the control group.

**Figure 3:**
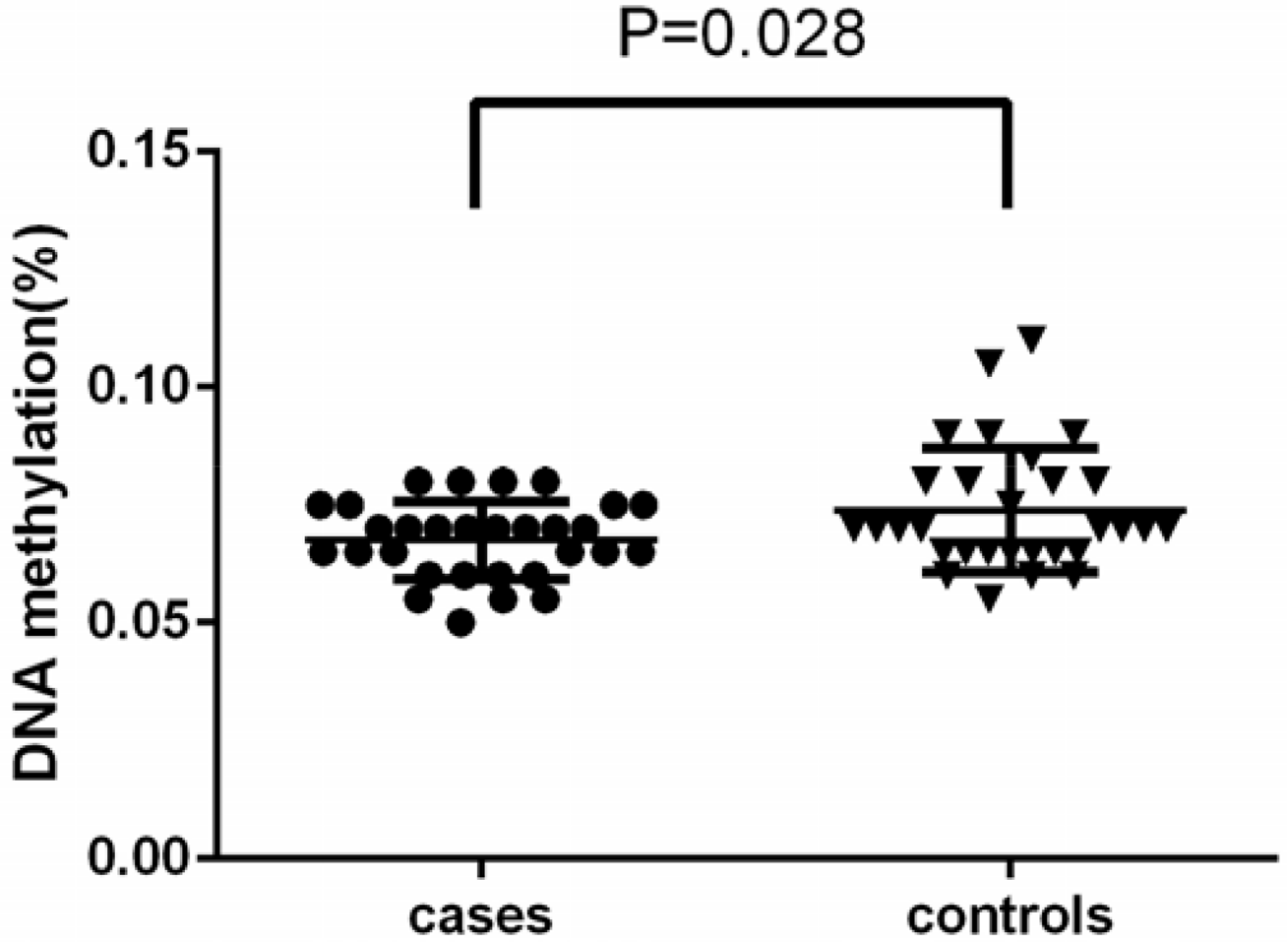
Comparison of the average methylation levels of CpG islands in the promoter region of TBC1D8 in osteoporosis and control groups

**Figure 4:**
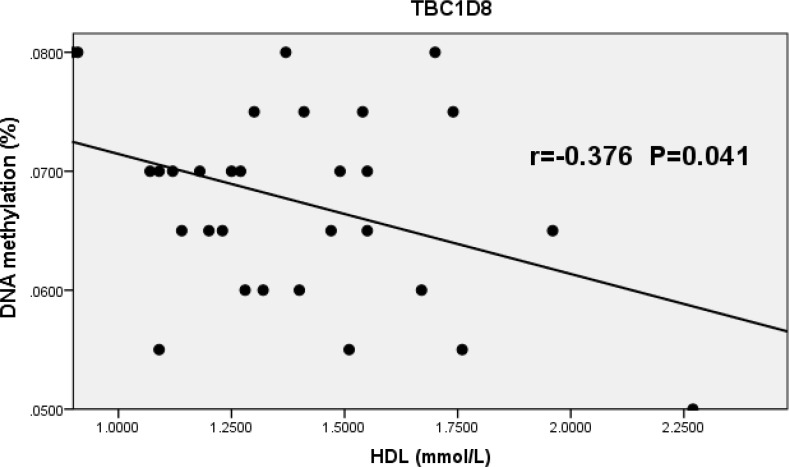
Correlation between the average methylation levels of CpG islands in the promoter region of TBC1D8 and HDL in osteoporosis.

The distributions of CpG island methylation in the promoter region of TBC1D8 in the osteoporosis and control groups is shown in Figure 1 and Figure 2. Representative samples are shown. The average CpG island methylation of the TBC1D8 gene was 6.75 ± 0.82 (%) in the osteoporosis group and 7.38 ± 1.30 (%) in the control group. These results were statistically significantly different (*P* = 0.028). Figure 3 shows the comparison. In addition, we subjected the methylation levels and biochemical variables to correlation analysis, the results of which are shown in Table 3 in detail. These results demonstrated that in the osteoporosis and control groups, correlations with methylation were associated with BMI, age of menopause, and calcium. Likewise, BMD of total hip and femur neck, and ALP, in both osteoporosis and control groups were all weakly negatively correlated with the TBC1D8 methylation level. We observed differences in the correlation between BMD and L1-L4, creatinine, uric acid, glucose, LDL, phosphorus, eGFR, P1NP and PTH. However, none of the correlations above were statistically significant. In the osteoporosis group; however, the CpG island methylation levels in TBC1D8 were negatively correlated with HDL (r = -0.376, *P* = 0.041) as shown in Figure 4.

**Table 3:** Correlation analysis between biochemical variables and gene TBC1D8 methylation in osteoporosis and control groups

Biochemical variables	Osteoporosis		Control	
	r value	*P* value	r value	*P* value
ALP	-0.308	0.098	-0.003	0.990
Creatinine	0.120	0.527	-0.285	0.127
Uric acid	-0.136	0.472	0.020	0.918
Glucose	-0.109	0.565	0.001	0.994
HDL	-0.376^*^	0.041^*^	-0.053	0.781
LDL	0.222	0.239	-0.100	0.600
Calcium	0.098	0.606	0.284	0.128
Phosphorus	-0.112	0.556	0.248	0.187
eGFR	-0.240	0.202	0.185	0.329
25 (OH) D	-0.032	0.866	-0.126	0.509
β-CTX	-0.310	0.095	-0.018	0..924
P1NP	-0.172	0.380	0.105	0.709
PTH	-0.080	0.687	0.003	0.991

^*^P < 0.05, it is significant statistical difference.

## DISCUSSION

The outcomes of this study demonstrate that participants in the osteoporosis group exhibited both lower BMI and shorter exercise times. Meta-analyses indicated that those with a BMI of 20 Kg/cm^2^ had a 95% increased risk of fracture compared to those with a BMI of 25Kg/cm^2^. Moreover, individuals with a BMI of 30Kg/cm^2^ had a 17% reduced risk of fracture compared to those with a BMI of 25 Kg/cm^2^ [18]. In addition, a lower BMI increased the risk of fracture in postmenopausal women. Studies indicated that the BMI per unit reduction was 1Kg/cm^2^, correlating to a 1.1-1.4 times increased risk of fracture [19, 20]. This BMI was indirectly associated with osteoporosis, and lower BMI negatively affected bone health. Albala *et al.* found that obesity was a protective factor for osteoporosis and that it reduced hormone binding globulin, increasing the level of free sex hormones. However, adjusted for the level of serum free sex hormones, obesity was directly correlated with BMD [21]. In addition, we observed that exercise was protective against osteoporosis, a result that is consistent with other findings [22]. With respect to sleep duration, participants in the control group averaged 6–7 hours of sleep per night and < 6 hours, but osteoporosis patients fell into two ranges, <6 hours or >7 hours. A previous study demonstrated that a long sleep duration may increase the risk for osteoporosis [23]. Our outcomes showed that more individuals in the osteoporosis group slept for longer periods of time (>7 h) compared with those in the control group. However, we also found that, compared with control group, a few of people in osteoporosis group also slept for a short time (<6 h), possibly because we included a limited number of participants.

There were significant differences between the osteoporosis and control groups regarding the comparison of BMD between L1-L4, the neck of the femur, and total hip. BMD in the osteoporosis group was statistically lower than in the control group. With respect to biochemical variables, individuals in the osteoporosis group had higher creatinine levels and thus higher eGFR levels compared with controls. Serum creatinine is an important indicator of renal function. The relationship between serum creatinine and osteoporosis is primarily manifested in the reduction of renal absorption, which can cause bone loss [24, 25]. Additionally, our results demonstrated that participants with osteoporosis had higher PTH, β-CTX, P1NP, and lower 25(OH)D, consistent with previous studies [26, 27]. PTH is a peptide hormone secreted by parathyroid glands that promotes intestinal calcium absorption and renal calcium reabsorption. Bone calcium stimulates osteoblast- and osteoclast-mediated release regulates calcium ion concentration in the body at a normal level. Research has shown that 25(OH)D is correlated with BMD [27, 28]; however, additional studies have also found that 25 (OH) D has no correlation with BMD [29, 30]. Generally speaking, our study found significant difference in the creatinine, eGFR, PTH, β-CTX and 25(OH)D levels between the osteoporosis and control groups.

A few studies that also examined genes related to osteoporosis described a potential link between gene methylation and osteoporosis. H. Lv. et al. [31] examined methylation of the ER-α gene in white blood cells in postmenopausal women and premenopausal women, and the ER-α gene methylation levels in postmenopausal women were significant higher than those in premenopausal women. However, the relationship between bone remodeling and regulation of ER-α expression remains unknown. Another study that investigated the ER-α gene found that methylation of this gene may influence its tissue-specific expression in human osteoblastic cells [32]. ApoE has also been associated with BMD at the level of gene polymorphisms [33, 34] and gene methylation [35]. However, the actual mechanism remains unclear and needs further exploration.

To our knowledge, the TBC1D8 gene has not been previously examined with respect to osteoporosis. It is a member of the human regulatory protein gene and a GTP activating factor. The Rho-GTP enzyme, which belongs to the GTP enzyme family, is a signal transduction molecule required for the maintenance of the cytoskeleton and formation of the fold edge, and activation of the Rho-GTP enzyme can increase osteoclast activity [36]. A genome wide analysis found that the TBC1D8 gene is closely associated with osteoporosis [11]. We found that the methylation level of the promoter region CpG islands in the TBC1D8 gene was significantly lower in those with osteoporosis compared to controls. With elevated methylation levels of TBC1D8, the GTP enzyme was activated to enhance osteoclast activity, resulting in osteoporosis. Zoledronic acid, a drug known to treat osteoporosis, inactivates the GTP enzyme by inhibition of cholesterol and lipid synthesis, inhibiting apoptosis of osteoclasts. TBC1D8 may participant in this regulation, but the specific mechanism remains unknown.

We conducted correlation analyses between gene methylation and biochemical variables/BMD. As shown in Table 3, we found that the methylation level of CpG islands in the promoter region showed a negative linear correlation with HDL in the osteoporosis group. However, the correlation was not reflected in LDL, nor in other factors. As mentioned before, the pharmacological mechanism of zoledronic acid occurs via inhibition of cholesterol and synthesis of diallyl lipids to inactivate the GTP enzyme and inhibit osteoclasts. TBC1D8 is an activated factor of the GTP enzyme, suggesting that TBC1D8 may be associated with lipoprotein. In addition, statins were found to be a protective factor for osteoporosis [37], consistent with our results. Further experiments are needed to determine specific mechanisms of these findings.

## MATERIALS AND METHODS

### Sample collection

We used questionnaires to collect data from Han Chinese women aged >60 years in two communities (Zhen-Ming community and Ying-Feng community, Haishu District, Ningbo). The questionnaire included age, age of menopause, hypertension history, diabetes history, fracture history, frequency of calcium intake, and history of smoking and drinking. No participants had metabolic diseases or a history of taking medications that affect bone metabolism. Ethics approval was obtained from the Ethics Committee of Ningbo First Hospital, China. The methods in this study were in accordance with relevant guidelines and the Declaration of Helsinki. All participants provided informed consent.

### Biochemical measurements

We obtained fasting blood samples from all individuals. Serum calcium levels (Ca), serum phosphorus levels (P), serum uric acid (SUA), serum alkaline phosphatase (ALP), serum creatinine, glucose (Glu) were all measured by kits from Roche Diagnostics. High density lipoprotein and low density lipoprotein were determined using kits from Olympus Co. Ltd. We also detected the bone turnover markers parathyroid hormone (PTH), tropocollagen type 1 N-terminal propeptide (P1NP) and β-crosslaps of type I collagen containing cross-linked C-telopeptide (β-CTX) using Kits from Roche Diagnostics. 25-hydroxyvitamin D_3_ [25(OH)D] was measured with kits from Meikang Biological Tech.

### BMD testing

BMD was tested by dual-energy X-ray absorptiometry (DXA) on a Lunar Prodigy GE densitometer (Lunar Corp, Madison, WI, USA). Lumbar spine (L1-L4), left femoral neck and total hip were all sampled during testing. Diagnosis of osteoporosis was based on the following criteria (WHO, 1994): Participants with a T score <-2.5 SD were classified as having osteoporosis; those with a T score < − 1.0 SD but > -2.5 SD were classified as having low bone mass; and those with a T score > − 1.0 SD were classified as normal. Body mass index (BMI) was calculated as height divided by the square of weight.

### DNA extracting and methylation sequencing

DNA was extracted from peripheral blood samples following routine methods (DNeasy Blood & Tissue, Qiagen). We measured the DNA concentration using Nanodrop 100 (Aosheng, Hangzhou city, China). Purified DNA was combined with sodium bisulfate DNA conversion chemistry (EpiTech, Bisulfite Kits, Qiagen). The DNA sequencing primers were designed by Methyl Primer Express (version 1.0), as shown in Table 4. Polymerase chain reaction (PCR instrument, Thermal Scientific Co. Ltd., Wilmington, USA) was utilized amplify the objective strips. DNA methylation was evaluated by applying the capture sequencing (Illumina Co. Ltd., San Diego, USA).

**Table 4:** The primers for DNA methylation assay

	Primer sequences
TBC1D8-1F	GTATTTYGTTGTTATTTGGTTTTA
TBC1D8-1R	CRAACTTATTTTCCTTTTTTCTAC
TBC1D8-2F	GTTTTTGGTTTTTTTTTAGTYGT
TBC1D8-2R	AAAACTTTTTCCCTATCCCT

### Statistical analysis

All statistical analyses were performed using the SPSS package (version 22.0) or Graghpad software (version 6.0). *T*-tests on two independent samples were used to investigate the differences of the TBC1D8 CpG island methylation levels between cases and controls. Linear regression was used to evaluate the association between the TBC1D8 CpG island methylation levels and biochemical variables in the cases and controls, respectively.

## CONCLUSIONS

Low BMI, reduced exercise time and variable sleep durations were associated with a higher risk of osteoporosis. In addition, high creatinine, low 25(OH)D, high β-CTX, and high PTH may increase osteoporosis risk. Low methylation levels of CpG islands in the promoter region of TBC1D8 were associated with osteoporosis in Chinese Han postmenopausal women, and a negative linear correlation was found between the TBC1D8 methylation level and HDL in women with osteoporosis.
